# Glial expression of Drosophila *UBE3A* causes spontaneous seizures that can be modulated by 5-HT signaling

**DOI:** 10.1016/j.nbd.2024.106651

**Published:** 2024-08-26

**Authors:** Saul Landaverde, Megan Sleep, Andrew Lacoste, Selene Tan, Reid Schuback, Lawrence T. Reiter, Atulya Iyengar

**Affiliations:** a Department of Biological Sciences, University of Alabama, Tuscaloosa, AL, United States of America; b Department of Neurology, University of Tennessee Health Science Center, Memphis, TN, United States of America; c Department of Anatomy & Neurobiology, University of Tennessee Health Science Center, Memphis, TN, United States of America; d Department of Pediatrics, University of Tennessee Health Science Center, Memphis, TN, United States of America; e Alabama Life Research Institute, University of Alabama, Tuscaloosa, AL, United States of America; f Center for Convergent Bioscience and Medicine, University of Alabama, Tuscaloosa, AL, United States of America

**Keywords:** Duplication 15q syndrome, UBE3A, Epilepsy, Drosophila models

## Abstract

Misexpression of the E3 ubiquitin ligase gene *UBE3A* is thought to contribute to a range of neurological disorders. In the context of Dup15q syndrome, additional genomic copies of *UBE3A* give rise to the autism, muscle hypotonia and spontaneous seizures characteristics of the disorder. In a Drosophila model of Dup 15q syndrome, it was recently shown that glial-driven expression of the *UBE3A* ortholog *dube3a* led to a “bang-sensitive” phenotype, where mechanical shock triggers convulsions, suggesting glial *dube3a* expression contributes to hyperexcitability in flies. Here we directly compare the consequences of glial- and neuronal-driven *dube3a* expression on motor coordination and seizure susceptibility in Drosophila.

To quantify seizure-related behavioral events, we developed and trained a hidden Markov model that identified these events based on automated video tracking of fly locomotion. Both glial and neuronal driven *dube3a* expression led to clear motor phenotypes. However, only glial-driven *dube3a* expression displayed spontaneous seizure-associated immobilization events, that were clearly observed at high-temperature (38 °C). Using a tethered fly preparation amenable to electrophysiological monitoring of seizure activity, we found glial-driven *dube3a* flies display aberrant spontaneous spike discharges which are bilaterally synchronized. Neither neuronal-*dube3a* overexpressing flies, nor control flies displayed these firing patterns. We previously performed a drug screen for FDA approved compounds that can suppress bang-sensitivity in glial-driven *dube3a* expressing flies and identified certain 5-HT modulators as strong seizure suppressors. Here we found glial-driven *dube3a* flies fed the serotonin reuptake inhibitor vortioxetine and the 5-HT_2A_ antagonist ketanserin displayed reduced immobilization and spike bursting, consistent with the previous study. Together these findings highlight the potential for glial pathophysiology to drive Dup15q syndrome-related seizure activity.

## 1. Introduction

Altered expression of the E3 ubiquitin ligase *UBE3A* in the nervous system is associated with a variety of neurological disorders (LaSalle et al., 2015; Lopez et al., 2019). In humans, *UBE3A* is located on 15q11.2-q13 and is paternally imprinted (Lalande, 1996; Vu and Hoffman, 1997). Maternal allele deletions and loss-of-function mutations in *UBE3A* cause Angelman syndrome (Kishino et al., 1997; Matsuura et al., 1997), characterized by cognitive impairment, developmental delay and a consistently happy demeanor (Angelman, 1965; Williams, 2005). In contrast, maternal duplications of the 15q11.2-q13 region (Dup15q syndrome), are associated with autism spectrum disorder (ASD) as well as intellectual disability, muscle hypotonia and pharmacoresistant epilepsy (Battaglia, 2008; DiStefano et al., 2016; Urraca et al., 2013). Indeed, as many as 3–5 % of all ASD cases are estimated to arise due to Dup15q syndrome (Depienne et al., 2009), and difficult to control seizures are a major concern for the families of patients with the disorder (Conant et al., 2014). Although many genes are located in the 15q11.2-q13 region, *UBE3A* is the only gene that is paternally imprinted (maternally expressed in neurons). Paternally derived duplications of 15q11.2-q13 may be associated with anxiety and sleep problems or show no phenotypes at all, but are rarely associated with epilepsy (Cook et al., 1997; LaSalle et al., 2015; Urraca et al., 2013). Given the promiscuity of UBE3A, and E3 ubiquitin ligase, in marking proteins for proteolytic degradation, the molecular pathways linking *UBE3A* overexpression to Dup 15q-associated phenotypes remain to be fully elucidated (reviewed in LaSalle et al., 2015).

To determine how *UBE3A* overexpression causes Dup15q syndrome, several mouse models have been created (Copping et al., 2017; Smith et al., 2011; Takumi, 2011). Mice carrying a duplication of a 6.3 Mb region syntenic to 15q11.2-q13 in humans display abnormal social interaction and behavioral inflexibility, but only when paternally inherited (Nakatani et al., 2009). Neuron-specific overexpression of *Ube3a* in mice is linked with increased anxiety-like behaviors, learning and memory deficits and hypersensitivity to chemoconvulsants (Copping et al., 2017). Although at least one model showed behavior differences after strong chemical seizure induction in mice expressing elevated *Ube3a* (Krishnan et al., 2017), none of the models thus far recapitulate the spontaneous seizure phenotypes characteristic of Dup15q syndrome. Studies in *Drosophila melanogaster* indicate that overexpression of *dube3a* (the ortholog of *UBE3A*) in glia, not neurons, results in a “bang-sensitive” phenotype (Hope et al., 2017). The bang-sensitive seizure assay (BSA) employs a mechanical shock to trigger stereotypic patterns of spasm and paralysis indicative of seizure activity in flies (Hope et al., 2017; Roy et al., 2020). This seizure-like activity is reminiscent of other fly mutants such as *FAK* (Ueda et al., 2008) and *zydeco* (Melom and Littleton, 2013) where glial disfunction is thought to contributed to behavioral phenotypes. Although the fly glial expression model of Dup15q syndrome recapitulates aspect seizure phenotypes using the BSA, a more unbiased and automated system is needed for larger anti-epileptic drug screening using this valuable disease model system. Furthermore, in flies expressing Dube3a in glia, direct electrophysiological observations of seizure activity would exclude potential neuromuscular confounds, providing a more rigorous interpretation of the bang-sensitive phenotype.

Here we employed an automated locomotion tracking system (*IowaFLI Tracker*, Iyengar et al., 2012) coupled with a newly-developed machine-learning approach to quantify seizure-related movement of flies overexpressing Dube3a in either glial cells or neurons. Using the same fly populations, we also investigated spontaneous seizure activity in animals expressing Dube3a in glia versus neurons using head fixed electrophysiology for the first time. Finally, we show that drugs previously shown to suppress epileptic activity in this model could also effectively suppress the spontaneous epileptic behavior.

## 2. Materials and methods

### 2.1. Drosophila stocks and husbandry

The UAS *dube3a* transgenic construct (BL 90375) pan-glial *repo*-Gal4 driver (BL 7415) and pan-neuronal *neurosynaptbrevin nSyb-Gal4* driver (BL 51941) have been previously described. All flies were reared on standard cornmeal media (Bloomington Stock Center Recipe) and kept at 25 °C (70 % relative humidity) on a 12:12 light-dark cycle.

### 2.2. Lifespan and behavioral analysis

Lifespans were performed as described in Iyengar et al. (2022). Flies were collected in a 1-day age-range under CO_2_ anesthesia and kept in standard vials. The number of survivors was counted daily, and flies were transferred to fresh food three times per week.

**Fig. 1.**
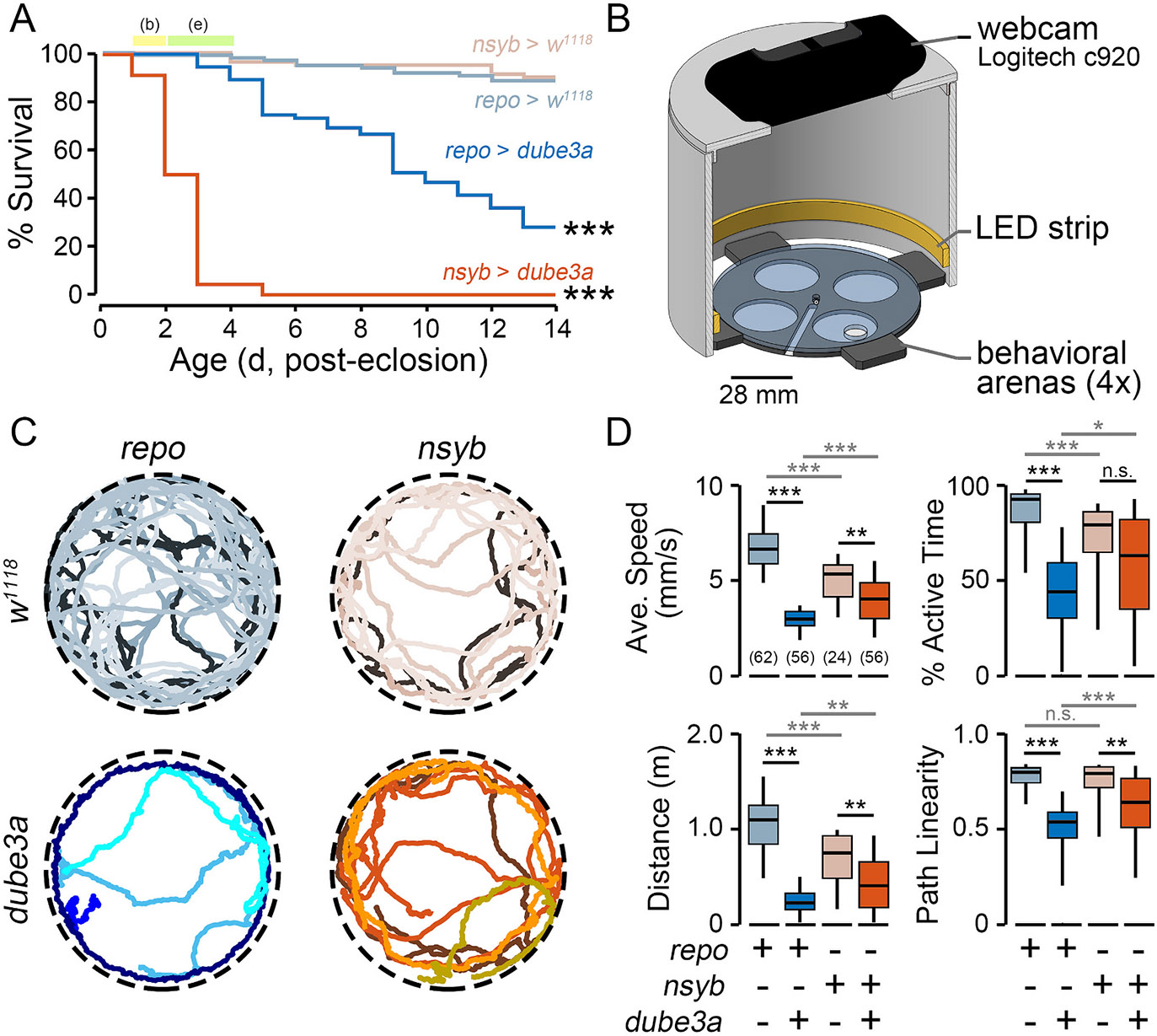
Survival and motor coordination phenotypes produced by *dube3a* overexpression. (A) Post-eclosion survival of *repo > dube3a, repo > w^1118^, nsyb > dube3a* and *nsyb > w*^*1118*^ flies. Flies aged 1–2 d were used for automated behavioral experiments (b), and flies aged 2–4 d were used for electrophysiological experiments (e). (B) Diagram of automated video tracking chamber. Up to four flies were loaded in each of the behavioral arenas. (C) Representative tracks (30-s duration) of four flies from the respective genotypes (temperature: 22 °C). (D) Distributions of locomotion characteristics over the 120-s observation period for the respective genotypes. Box plots indicate the 25th, 50th, and 75th %-tiles; whiskers indicate the 5th and 95th %-tiles. Sample sizes indicated in parentheses in the average speed panel. For lifespan analysis *dube3a* overexpression populations were compared against respective *w*^*1118*^ controls (log-rank test). For locomotion analysis, a Kruskal Wallis non-parametric ANOVA (Bonferroni-corrected rank-sum post hoc test) was employed. * *p* ≤ 0.05; ** *p* ≤ 0.01; *** *p* ≤ 0.001.

Automated open-field behavior monitoring was performed as described in (Iyengar et al., 2012), with modifications for heating the arena (Fig. 1B). Polyacrylate behavioral arenas were placed on a piece of Whatman #1 filter paper, which was in-turn placed on the Peltier temperature-controlled stage (AHP-1200CPV TECA Corporation, Chicago, IL). Arena temperature was monitored by a T-type thermocouple (5SRTC-GG-T-30-36, Omega Engineering, Norwalk, CT) connected to a data acquisition card (NI TC-01, National Instruments, Austin TX). Stage temperature was controlled by a custom-written LabVIEW script (National Instruments). A custom-built PVC lighting cylinder with an LED strip was placed above the behavioral arenas (see Ueda et al., 2023 for details). Light intensity (~ 1000 lx) was set by a current controller driving LEDs (~100 mA).

**Fig. 2.**
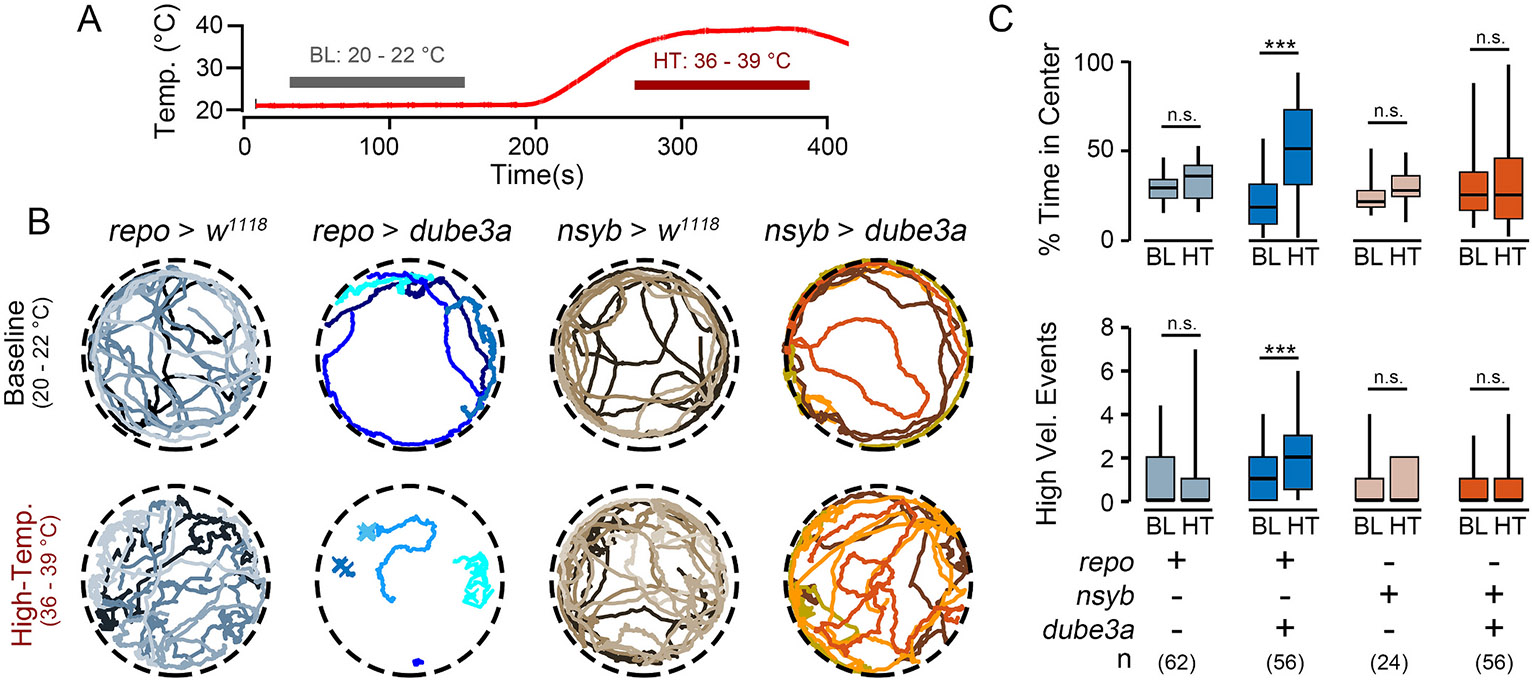
Motor phenotypes induced at high temperature in glial- and neuronal-driven *dube3a* overexpression. (A) Temperature profile of the behavior arena during the experimental protocol. (B) Representative tracks (30-s) from *repo > dube3a, nsyb > dube3a* and respective controls during baseline activity (20–22 °C) and activity at high-temperature (36–39 °C). Note the abrupt and uncoordinated activity in *repo > dube3a* flies. (C) Quantification of time in center (upper panel) and high velocity events (lower panel) during the baseline and high-temperature periods. Note the increased values at high-temperature in *repo > dube3a* flies. Significance determined by Kruskal Wallis non-parametric ANOVA, followed by Bonferroni-corrected post hoc tests. (* p ≤ 0.05; ** p ≤ 0.01; *** p ≤ 0.001).

For open-field behavior experiments, four flies of a selected genotype and sex were placed without anesthesia into behavior arenas. We employed a standardized 10-min protocol (Fig. 2A) to monitor fly activity: 180 s at baseline temperature (21 °C), temperature-ramp to 36 °C (90 s), high-temperature period (36 °C- 39 °C, 120 s), return to baseline (210 s). Selected 2-min periods during the baseline and high-temperature phases (30–150 s and 270–390 s) were chosen for subsequent analysis. Video recordings were made with a Logitech c920 webcam (frame rate: 30 fps) controlled by a LabVIEW script. Fly locomotion videos were analyzed using IowaFLI Tracker (Iyengar et al., 2012) running on MATLAB (R2023b, Mathworks, Natick, MA).

IowaFLI Tracker captures several locomotion parameters including average speed, percent active time, and total distance traveled as described in Iyengar et al. (2012). The path linearity measure is described in Chi et al. (2019), while the percent time in center is described in Kaas et al. (Kaas et al., 2016). High velocity events (Fig. 2) were computed by finding the number of times a fly’s speed exceeded 10 SD greater than the average speed of that fly in the recording. All computations were done in MATLAB (R2023b).

Identification of “posture loss” and “convulsions” (Fig. 2F) required manual scoring. Posture loss was operationally defined as a fly on its back (supine) or side or otherwise not standing on its legs. Convulsions were operationally defined as a fly displaying a wing buzz or otherwise abruptly moving in the arena while not walking. Behaviors were scored offline by a trained observer blinded with respect to the genotype.

### 2.3. Behavioral classifier construction

Full details of the immobilization behavior classifier can be found in the Supplemental Methods. This classifier consists of a hidden Markov model (HMM) that classifies each 2-s window of movement into one of three states: “walking”, “pausing” and “immobilization” in the activity pattern of a fly. The analysis consists of three stages: 1) Pre-processing, where the full x-y trajectory of the fly is split into sequential short trajectories (2 s) which are then aligned and scaled according to principal components. 2) Classifier construction, where we created an HMM based on a training data set to determine one of three states, ‘walking’, ‘pausing,’ and ‘immobilization’, was most likely for a particular 2 s trajectory. The training set consisted of all *repo > w*^*1118*^ and *repo > dube3a* videos collected (total *n* = 253 flies, 136,620 trajectories). 3) Decoding, where the HMM is used to classify trajectories from the full data sets into one of the three categories. All code was implemented using the MATLAB Statistics toolbox.

### 2.4. Tethered fly electrophysiology

Electrophysiological analysis of seizure activity was based on methods described in Iyengar and Wu (2014). Flies were anesthetized on ice and fixed to a tungsten tether pin with UV-cured cyanoacrylate glue (Loctite #4311). Following a ~ 15 min recovery period, sharpened tungsten electrodes were inserted into the top-most dorsal longitudinal flight muscles (DLMa) with a similarly constructed reference electrode inserted into the dorsal abdomen. Muscle action potentials were amplified by an AC amplifier (AM Systems Model 1800) and digitized by a DAQ card (NI USB 6210) controlled by a custom-written LabVIEW script. Spike trains were analyzed off-line by previously described approaches implemented in custom-written MATLAB scripts.

Identification of burst discharges was performed as described in Lee et al., (Lee et al., 2019). The instantaneous firing frequency (${\text{ISI}}^{-1}$) for each spike was defined as the reciprocal of the inter-spike interval between the current spike and succeeding spike. The instantaneous coefficient of variation, CV_2_, for a pair of ${\text{ISI}}^{-1}$ values i and i+1 was $2^{⋆}|{\text{ISI}}_{i}^{-1}\text{-}{\text{ISI}}_{i+1}^{-1}|∕({\text{ISI}}_{i}^{-1}+{\text{ISI}}_{i+1}^{-1})$. Smaller CV_2_ values indicate rhythmic firing spike trains, while higher CV_2_ values indicate irregular firing. In plots of the instantaneous firing frequency versus CV_2_, bursts are readily observed as loops in the trajectory switching from burst firing (low CV_2_) to inter-burst firing (high CV_2_). A custom-written MATLAB script counted the number of loops in these trajectories to report the number of bursts.

### 2.5. Pharmacology

**Fig. 3.**
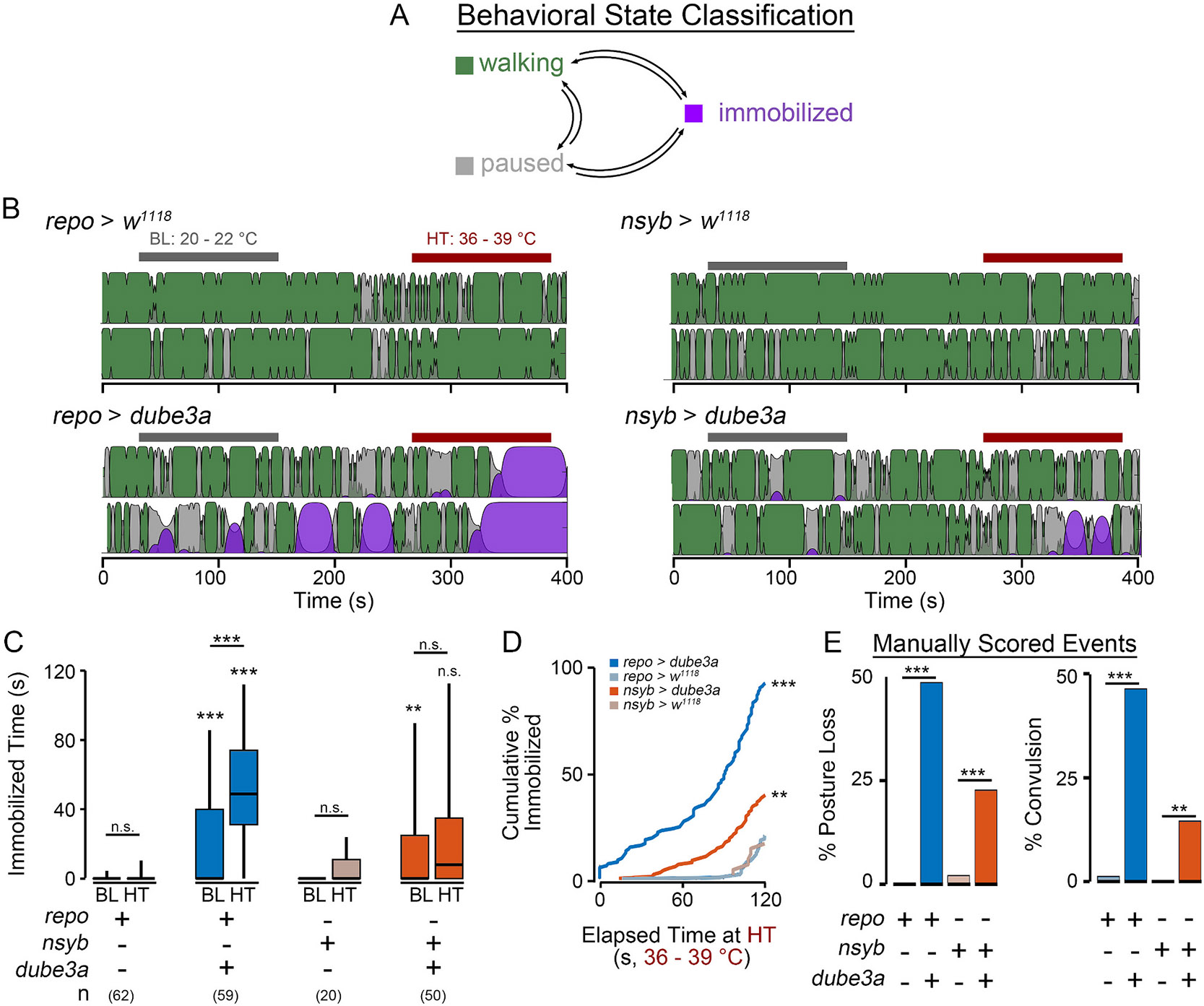
Identification of seizure-related behavioral states based on hidden Markov classification. (A) Illustration of a hidden Markov classifier (HMC) designed to identify three behavioral states: walking, paused and immobilized based on fly locomotion trajectories. Arrows indicate transitions between behavioral states. (B) Representative classification of activity by the HMC of respective genotypes (activity from two flies is classified for each genotype). Colors indicate the classified state (walking-green, paused-pink, immobilized-purple), and the height represents the confidence of classification ranging from 0 (low confidence) to 1 (high confidence). Bars above indicate baseline (BL) and high-temperature (HT) periods. (C) Distributions of total immobilization time during the baseline and high-temperature periods. (D) Cumulative percentage of flies classified as immobilized during the high-temperature period as a function of elapsed time. (F) Manual scoring of posture loss and convulsions during the high-temperature period. For panel C significance determined by Kruskal Wallis non-parametric ANOVA (Bonferroni-corrected post hoc). For panel E, log-rank test comparing *repo > dube3a* or *nsyb > dube3a* expression vs *w*^*1118*^ controls. For panel F, Fisher’s exact test. (* p ≤ 0.05; ** p ≤ 0.01; *** p ≤ 0.001).

To block nicotinic acetylcholine receptor (nAChR)-mediated neurotransmission (Fig. 3H), we used the nAChR antagonist mecamylamine (Sigma M9020). Mecamylamine was injected into the dorsal vessel (analogous to the mammalian heart) using previously described methods (Lee et al., 2019) the tethered fly preparation.

The 5-HT_1A_ and SERT agonist vortioxetine (VTX, HY-15414) and the 5-HT_2A_ antagonist ketanserin (KET, HY-10562) were obtained from medchemexpress.com. For drug feeding experiments, VTX and/or KET were first dissolved in a DMSO stock solution (40 mM) marked with 2.5 % (w/vol) blue #1 dye. To make drug-laced media, stock solution was diluted to final concentration (0.4 μM or 0.04 μM) into melted fly food mix. Drug-fed flies were reared on VTX- or KET-laced media from larval hatching.

### 2.6. Statistical analysis

All statistical analysis was done in MATLAB using the Statistics Toolkit. A log-rank test was used to compare lifespan curves (Fig. 1A), and Fisher’s exact test was used to compare relative fraction of flies displaying posture loss or convulsions (Fig. 2F). All other statistical comparisons were done using non-parametric Kruskal-Wallis ANOVA following by a rank-sum post hoc test. *P*-values were corrected using the Holm-Bonferroni method. A complete list of all statistical comparisons is listed in Supplemental Table 1.

## 3. Results

### 3.1. Distinctions in survival of glial vs. neuronal overexpression of dube3a

Previous studies in Drosophila indicated that glial, but not neuronal, overexpression of *dube3a* resulted in strong bang-sensitive phenotypes (Hope et al., 2017). Disfunction in both neuronal and glial physiology have been implicated in Dup15q syndrome, so distinguishing the specific etiological contributions of glial or neurons is critical to the development of an effective model of the disease. To directly compare the consequences of *dube3a* overexpression in neurons versus glia, we used the Gal4-UAS system to drive expression of UAS-*dube3a* under the control of the pan-glial driver *reversed polarity*-Gal4 (*repo > dube3a*) or the pan-neuronal driver *neurosynaptobrevin*-Gal4 (*nsyb > dube3a*). We compared these flies with the respective drivers crossed with a *w*^*1118*^ control strain (*repo > w*^*1118*^, and *nsyb > w*^*1118*^) which approximates the genetic background but lacks *dube3a* overexpression.

All crosses produced viable adult offspring. Survival of the progeny was observed over a 14-d window (Fig. 1A). Overexpression *dube3a* in either glia or neurons resulted in clear survival phenotypes. The median lifespan of *repo > dube3a* flies was 10 d, while *nsyb > dube3a* hand a median lifespan of only 2 d, significantly shorter than their glial expressing counterparts (p ≤ 0.001). Both overexpression populations displayed greater mortality compared to respective control flies (*repo > w*^*1118*^ or *nsyb > w^1118^*) over the 14-d window. Thus, to ensure sufficient sample sizes, 2 d-old flies were used for behavior experiments, and 3-4 d-old flies for electrophysiological analysis. Survival analysis of glial and neuronal-driven overexpression of *dube3a* indicates that chronic overexpression of *dube3a* is pathological in both glia and neurons.

### 3.2. Glial overexpression of dube3a causes gross motor defects

To characterize behavioral correlates of *dube3a* expression in glial and neurons, walking activity was observed for *repo > dube3a* and *nsyb > dube3a* flies in an open arena (Fig. 1B). Activity was recorded via webcam and IowaFLI Tracker tracked the positions (x- and y-coordinates) of each fly to quantify characteristics of locomotion. Both *nsyb > dube3a* and *repo > dube3a* flies displayed detectable differences in walking compared to controls (Fig. 1C, Supplemental Video 1). However, motor phenotypes were most extreme in *repo > dube3a* individuals. Over a 3-min observation period, *repo > dube3a* females displayed clear reductions in average speed (54.8 %), active time (52.3 %), total distance traveled (79.2 %) compared to *repo > w*^*1118*^ controls. Although *nsyb > dube3a* females also displayed reductions in these parameters compared to *nsyb > w*^*1118*^ controls, the effect sizes were relatively smaller (average speed: 24.3 %, active time: 20.4 %, distance traveled, 45.7 %), and in the case of active time were not statistically significant.

Interestingly, unlike *repo > w*^*1118*^ controls which largely displayed straight-line or gently curved locomotion, *repo > dube3a* animals often displayed ‘jittery’ trajectories (Fig. 1C). To quantify this feature, we developed a measure called *path linearity* which is the average ratio of displacement to distance traveled over 2-s time windows (Fig. 1D). The path linearity of *repo > dube3a* flies was markedly reduced compared to *repo > w*^*1118*^ controls (median: 0.53 vs 0.79, *p* ≤ 0.001), while *nsyb > dube3a* flies displayed an intermediate effect compared to their controls (median: 0.64 vs 0.79, p ≤ 0.001). Together, these findings indicate glial expression of *dube3a* and, to a lesser extent, neuronal *dube3a* overexpression led to disruptions in motor coordination.

### 3.3. Heat-induced and spontaneous seizure-related behavior in repo>dube3a

Prior studies report seizure-like activity in *repo > dube3a* flies can be induced by mechanical shock (vortexing), heat or visual stimulation (Hope et al., 2017). To further study this seizure-like behavior in open field arenas, we used heat to trigger the activity as it avoided the mechanical artifacts associated with vortexing. In the open field arenas, *repo > dube3a,* and *nsyb > dube3a* flies (along with control flies) were monitored at the baseline temperature (21 °C) and then subjected to a 2-min period of high temperature stress (Fig. 2A, 36–39 °C). A Peltier stage below the open field arena controlled the temperature. Consistent with previous reports, at high-temperature, *repo > dube3a* flies displayed striking seizure-associated behaviors: “wing buzzing” and “spinning” events, followed by a period immobilization, where flies would twitch or otherwise make small movements, but not walk (Fig. 2B, Supplemental Video 2). In contrast, these behaviors were rarely observed in *nsyb > dube3a* flies and were absent in either the *repo > w^1118^* or *nsyb > w*^*1118*^ controls.

To quantify seizure-associated behaviors, activity was compared at the baseline temperature versus high temperature using several established measures of hyperexcitable behaviors (Fig. 2C). In open arenas, most flies tend to walk along the walls and spend little time in the arena center (‘thigmotaxis’, Besson and Martin, 2005), however certain hyperexcitable seizure-prone mutants such as *Shudderer* show increased time in the arena center (Kasuya et al., 2019). At baseline temperatures, both *repo > dube3a* and *nsyb > dube3a* flies (along with the control flies) spent relatively little time in the arena center. However, at high temperatures, *repo > dube3a* flies spent considerably more time in the center of the arena (*p* ≤ 0.001). Neither *nsyb > dube3a* flies nor the control flies displayed this increase. Similarly, there was an increase in the number of high-velocity events (corresponding with “wing-buzz” events) during high temperature in *repo > dube3a* flies, but not in the *nsyb > dube3a* or the control flies (p ≤ 0.001). Thus, glial overexpression of *dube3a,* but not neuronal expression, causes high-temperature dependent motor phenotypes reminiscent of seizure-like behavior.

### 3.4. Developing a machine-learning classifier to detect seizure-associated behaviors in flies

To establish an automated approach to identify specific moments of seizure-associated activity in *repo > dube3a* flies, we developed a machine-learning strategy. The behavioral presentation of high temperature-induced seizures in *repo > dube3a* is quite variable, with some flies displaying “spin” or “wing buzz” events, while others do not show clear “convulsion” events. However, in all cases, *repo > dube3a* flies display a prolonged period of immobilization, with leg-twitching and postural changes, but minimal movement (Supplemental Video 2). In contrast, control *repo > w^1118^* and *nsyb > w^1118^* flies either walk or engage in brief pauses in locomotion and quickly transition back to walking throughout the high-temperature period. We sought to distinguish and identify immobilization events from the normal patterns of walking interspersed with pauses which flies exhibit.

We developed a hidden Markov classifier (HMC) to identify ‘immobilization’ based on the fly’s positional trajectory. The HMC classifies a fly as ‘walking’ (high forward velocity), ‘paused’ (brief period of little forward movement) or ‘immobilized’ (prolonged period of little forward movement) in each video frame based on the fly’s locomotion characteristics at a particular moment, along with the prior classified states (Fig. 3A). Control *repo > w*^*1118*^ and *nsyb > w*^*1118*^ flies were never classified as immobilized during normal locomotion at baseline temperatures. Details on the construction and parameters of the HMC can be found in the Supplemental Methods section.

As shown in Fig. 3B-D, during high-temperature stress, the HMC often classified *repo > dube3a* flies as immobilized (median immobilization time: 49 s), with nearly all animals (92 %) classified as immobilized for at least 2 s during the high temperature period. In contrast, most *nsyb > dube3a* flies were active during the temperature stress period, with only occasional immobilization events (median: 10 s, 40 % of flies). Importantly, repo>*w^1118^* and *nsyb > w*^*1118*^ control flies rarely displayed immobilization events (16 % and 19 % of flies) with a median of only a few frames (< 30, 1-s) marked as ‘immobilized’.

Direct observation of fly behavior in individuals overexpressing *dube3a* largely corroborated findings from the HMC (Fig. 3E). Scorers blinded to the genotype indicated ~49 % of *repo > dube3a* flies lost posture during high temperature stress (vs 0 % of *repo > w*^*1118*^ control flies). Similarly, 48 % of repo*>dube3a* flies displayed a ‘convulsion’ (defined as a wing buzz, spinning or caroming behavior, Supplemental Video 2). Control *repo > w*^*1118*^ flies rarely displayed this behavior (< 1 %). Consistent with the HMC findings, *nsyb > dube3a* flies had a mild phenotype, with 22.4 % displaying posture loss (vs 1.9 % for control flies) and 14.7 % displaying convulsions (vs. 0 % for control flies). Together these observations indicate the HMC approach can reliably identify moments of immobilization associated with high-temperature stress, and the reported values are consistent with manual observation of seizure-associated behavior in *repo > dube3a* individuals.

### 3.5. Electrophysiological analysis of seizure activity in glial versus neuronal dube3a overexpressing flies

**Fig. 4.**
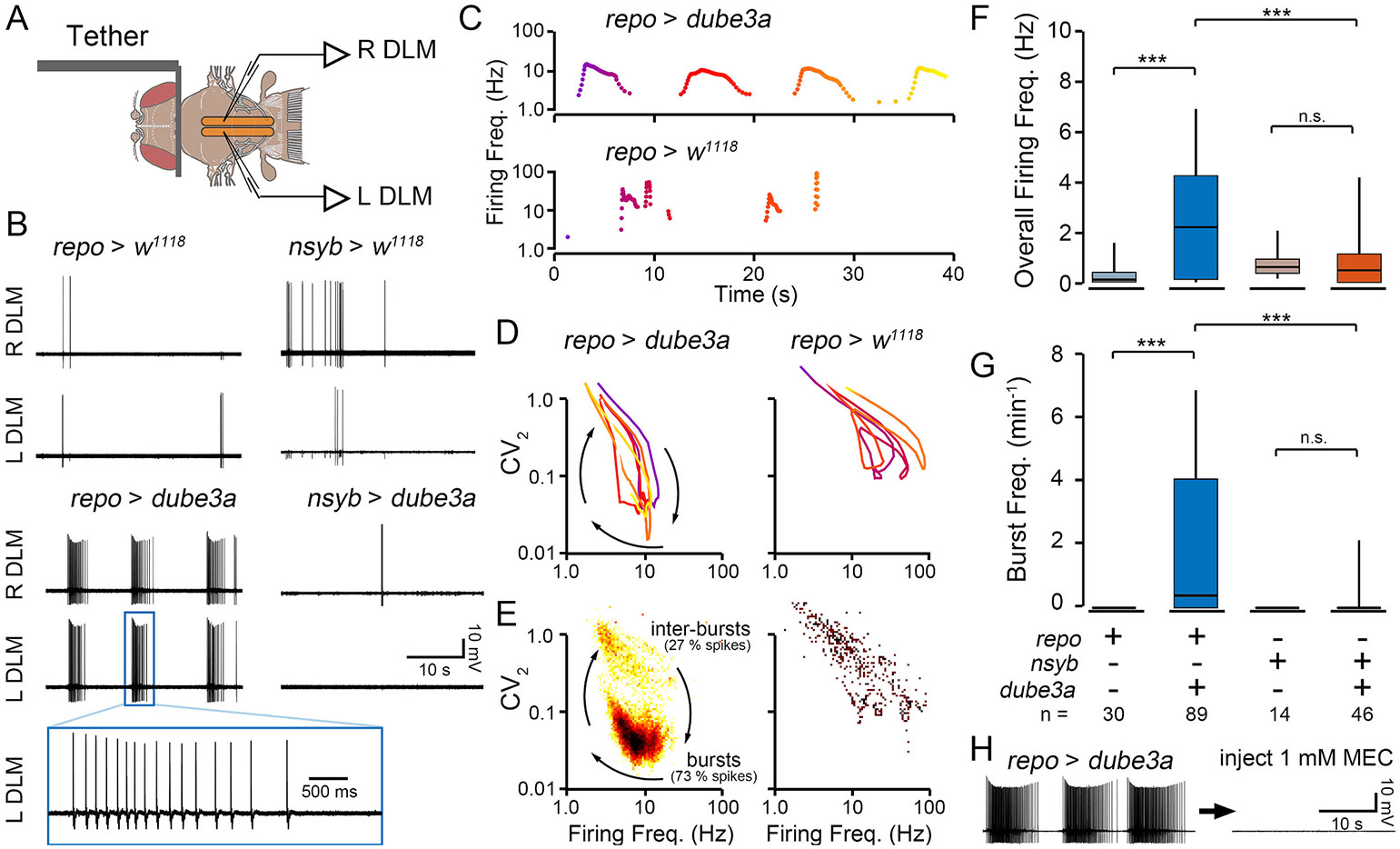
Electrophysiological monitoring of seizure activity in flies overexpressing *dube3a* in glia or neurons. (A) Illustration of the tethered fly preparation. Sharpened tungsten electrodes inserted into the left and right flight muscles (L DLM and R DLM) pick up spikes (see also Supplemental Video 3). (B) Representative traces of DLM flight muscle spiking from *repo > dube3a, repo > w*^*1118*^*, nsyb > dube3a* and *nsyb > w*^*1118*^ flies. Note the regular bursts discharges in *repo > dube3a* that are synchronized between the left and right muscle fibers. A selected spike burst is enlarged below. Spiking in the other genotypes is associated with grooming activity. (C) Representative plots of the instantaneous firing frequency of spiking in *repo > dube3a* and *repo > w*^*1118*^ flies. Color indicates elapsed time. (D) Distributions of the overall firing frequency. Sample sizes indicated above box and whisker plots. (E) Plots of the instantaneous firing rate versus instantaneous coefficient of variation (CV_2_) for the spike trains in (C). Lower CV_2_ values indicate rhythmic spiking, while higher CV_2_ values correspond with irregular firing. Note the oscillatory trajectory in *repo > dube3a,* with each loop corresponding to a single burst. (F) Bivariate histogram of firing trajectories in *repo > dube3a* and *repo > w*^*1118*^ flies. Color indicates the number of spikes at a particular firing frequency vs. CV_2_ value. Note the large number of spikes corresponding with bursts in *repo > dube3a*. (G) Quantification of the burst frequency in the respective genotypes. Bursts were not observed in the control genotypes, and only occasionally in the *nsyb > dube3a* flies. (H) Representative trace of a *repo > dube3a* fly before (left) and after (right) dorsal vessel injection of the nAChR blocker mecamylamine. Note the complete cessation of bursting activity. For (F) and (G), Kruskal-Wallis ANOVA, rank-sum post hoc test. (* p ≤ 0.05; ** p ≤ 0.01; *** p ≤ 0.001).

Given the clear seizure-like behavior of flies overexpressing *dube3a* in glia, we wanted to determine if more subtle, quantifiable, electrophysiological activity could be detected in these flies. We employed a tethered fly preparation previously utilized to characterize seizure activity (Iyengar and Wu, 2014). In an intact behaving fly, electrodes inserted in the dorsal longitudinal flight muscles (DLMs) enable prolonged recording of action potentials with minimal muscle damage (Fig. 4A, Supplemental Video 3). In wild-type flies, DLMs display characteristically rhythmic firing during flight and arrhythmic firing during grooming behavior (Lee et al., 2019). In seizure-prone flies, a wide array of aberrant mutant-specific firing patterns have been reported (Chi et al., 2022; Kaas et al., 2016; Lee and Wu, 2002; Pavlidis and Tanouye, 1995). Importantly, DLM spiking during seizure events is correlated with hypersynchronized activity across the brain as revealed by local field potential recordings (Iyengar and Wu, 2021).

In the tethered preparations, spontaneous DLM activity was monitored in flies overexpressing *dube3a* in either glia or neurons and the relevant controls (Fig. 4B). In control *repo > w*^*1118*^ and *nsyb > w*^*1118*^ occasional firing was identified which was correlated with bouts of grooming (Supplemental Video 3). The instantaneous firing frequency during the sparse bouts (< 1 s) of irregular spiking approached 100 Hz (e.g. Fig. 4C), largely consistent with previous reports of grooming-related firing (Lee et al., 2019). In sharp contrast, *repo > dube3a* flies often exhibited sustained bursts of rhythmic spiking (5–15 s), with instantaneous firing frequencies during these bursts reaching up to 20 Hz (Fig. 4C). These bursts were synchronized across the left and right muscles. Across the 240 s recording period, the overall DLM firing frequency of *repo > dube3a* flies was significantly higher than the *repo > w*^*1118*^ control individuals (Fig. 4F, p ≤ 0.001). Notably, the average firing frequency of *repo > dube3a* flies appeared to increase with age, although the effect was far from statistically significant given the sample size (Supplemental Fig. 1, r^2^ = 0.017, *p* = 0.33). Overexpression of *dube3a* in neurons (*nsyb > dube3a*) did not lead to spontaneous burst discharges (Fig. 4B). Instead, these flies displayed occasional grooming-related spiking, much like their *nsyb > w*^*1118*^ control counterparts (Fig. 4B). Indeed, no significant differences were observed in the overall firing frequency between *nsyb > dube3a* individuals and the *nsyb > w*^*1118*^ control flies (Fig. 4F).

To quantify characteristics of burst patterning in *repo > dube3a* flies, we constructed phase plots of the instantaneous firing frequency versus the instantaneous coefficient of variation, a measure of firing rhythmicity (CV_2_, Lee et al., 2019). These plots readily differentiate the irregular grooming-associated spiking in *repo > w*^*1118*^ flies with relatively high CV_2_ values from seizure-related burst discharges in *repo > dube3a* flies during which low CV_2_ values indicate rhythmic firing (Fig. 4D). Bivariate histograms of firing frequency vs CV_2_ from *repo > dube3a* spiking indicated stereotypic firing frequencies (~7 Hz) and CV_2_ values (~0.04) corresponding with bursting (Fig. 4E). Spiking trajectories in *repo > w*^*1118*^ flies did not approach this region in the firing frequency – CV_2_ plots. Thus, we counted the number of times the spiking trajectory entered or exited the bursting region to quantify the frequency of burst spike discharges. Compared to *repo > w*^*1118*^ counterparts, we found spike bursts in *repo > dube3a* flies occurred much more frequently (*p* ≤ 0.001), with a wide range of burst frequencies (Fig. 4G). Although bursting was occasionally observed in *nsyb > dube3a* flies (2/11 flies), across the cohort, there was no appreciable difference in the frequency of burst discharges from control *nsyb > w*^*1118*^ flies.

A hallmark of seizures is hypersynchronization of neuronal activity. As shown in Fig. 4B, in *repo > dube3a* flies, DLM spike bursts were synchronized between the left and right muscle fibers. This synchronization suggests spike bursts are centrally generated rather than arising through increased motor nerve or muscle excitability. To confirm the central origin of these bursts, we blocked central excitatory neurotransmission. Acetylcholine is the central excitatory neurotransmitter in flies, and the nicotinic acetylcholine receptor antagonist mecamylamine blocks central neurotransmission (Chi et al., 2022). We found injection of mecamylamine in *repo > dube3a* flies effectively suppressed bursting activity (Fig. 4H). Together, these observations indicate centrally generated hyper-synchronous activity drives spontaneous spike discharges in *repo > dube3a* flies.

### 3.6. Modulation of 5-HT signaling attenuates seizure activity in flies overexpressing dube3a in glia

In a previous screen for compounds ameliorating bang-sensitivity in *repo > dube3a* flies we uncovered several modulators of serotonin (5-HT) neurotransmission which significantly shorten the recovery time following mechanical shock (Roy et al., 2020). In fact, Dube3a can regulate monoamine synthesis through transcriptional regulation of GTP cyclohydrolase I (Ferdousy et al., 2011) and in mouse models of UBE3A related syndromes, Ube3a can modulate 5-HT levels in some regions of the brain (Farook et al., 2012). Elevation of overall 5-HT levels, 5-HT_1A_ receptor agonists or antagonists of 5-HT_2A_ receptors reduce recovery time in *repo > dube3a* flies, while 5-HT_1A_ antagonists and 5-HT_2A_ agonists increase recovery time of *repo > dube3a* flies (Roy et al., 2020). We selected two representative drugs from the previous screen, ketanserin and vortioxetine, to determine if either compound could attenuate spontaneous and heat-induced seizure-associated behavior in *repo > dube3a* flies. Ketanserin is a 5-HT_2A_ antagonist with a relatively strong effect accelerating recovery from mechanical shock, while vortioxetine is a selective 5-HT reuptake inhibitor (SSRI) with a milder but still significant recovery effect.

**Fig. 5.**
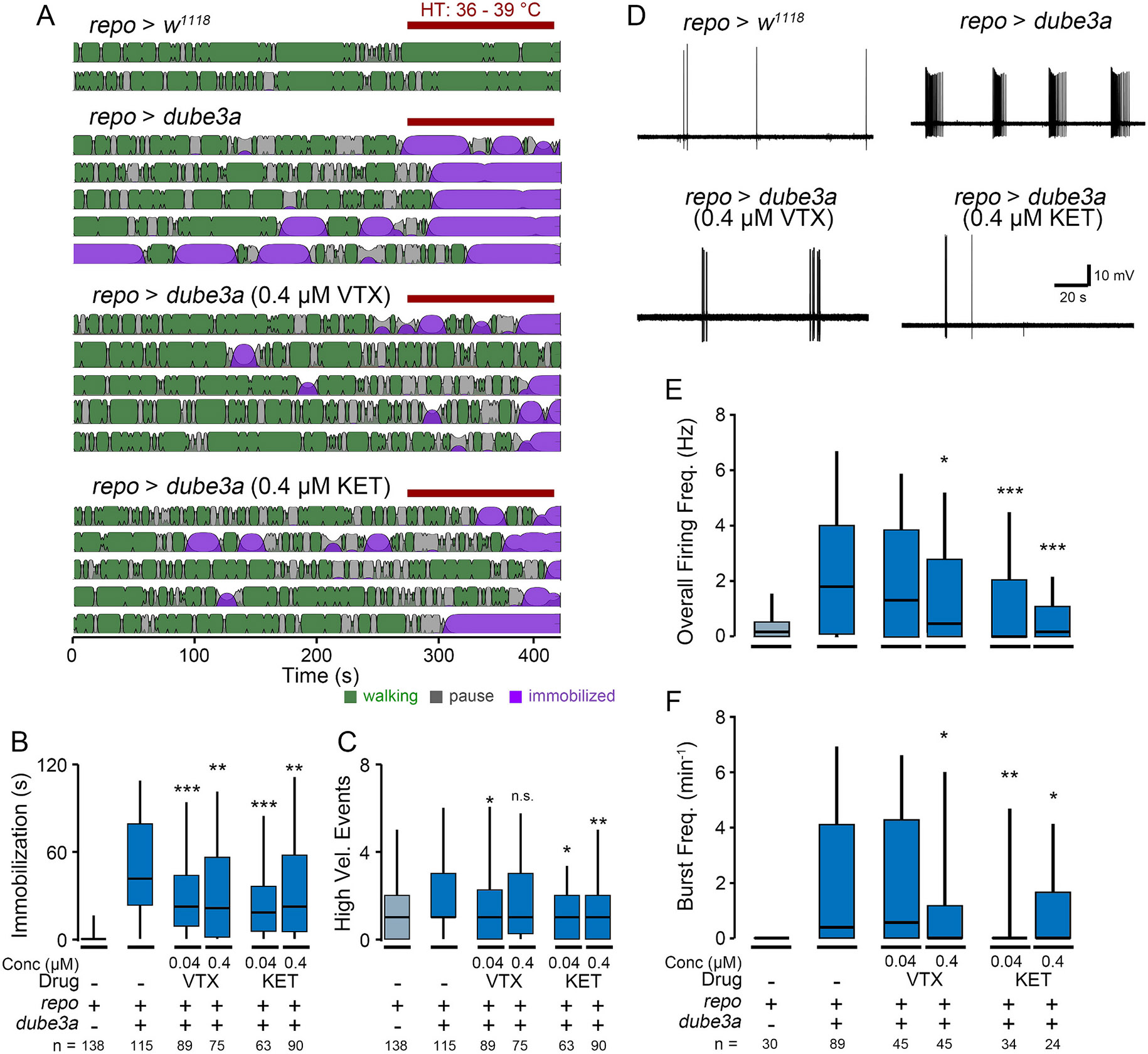
Modulation of seizure-associated behaviors and spike burst discharges in *repo > dube3a* flies with vortioxetine and ketanserin. (A) Representative activity patterns as determined by HMC in control-diet *repo > w*^*1118*^ flies, and *repo > dube3a* flies fed either control diet, vortioxetine (VTX, 0.4 μM) or ketanserin (KET, 0.4 μM). Plots constructed as in Fig. 2C, red bar indicates high-temperature period. (B—C) Box plots of (B) immobilization time and (C) high velocity events during the high-temperature period in the respective groups. (D) Representative traces of DLM spiking in *repo > dube3a* flies treated with VTX or KET compared to *repo > dube3a* and *repo > w*^*1118*^ flies reared on the control diet. *(E*-F) Quantification of (E) overall firing frequency and (F) burst frequency in the respective groups. Sample sizes as indicated, statistical significance determined by Kruskal-Wallis ANOVA, rank-sum post hoc test vs the respective control-fed *repo > dube3a* group. (* p ≤ 0.05; ** p ≤ 0.01; *** p ≤ 0.001).

We found *repo > dube3a* flies fed either vortioxetine or ketanserin were resistant to high-temperature stress compared to controls on non-drug food. Specifically, during the high-temperature period of the video tracking protocol, *repo > dube3a* flies fed either drug at concentrations of 0.04 μM or 0.4 μM were immobilized for less time than control flies (Fig. 5A-B). In several cases, the rate of high-velocity events was reduced as well (Fig. 5C, 0.04 uM vortioxetine, 0.04 or 0.4 μM ketanserin). Furthermore, even at baseline temperature, spontaneous immobilization events in *repo > dube3a* flies were reduced in animals raised on vortioxetine or ketanserin food (Supplemental Fig. 2). Notably, neither drug completely reverses the hyperexcitability phenotypes, as immobilization and high velocity events occurred at a higher frequency in drug-treated *repo > dube3a* individuals compared to *repo > w*^*1118*^ control flies. Together, these observations are consistent with findings from the previous BSA study, where SSRIs and 5-HT_2A_ antagonists attenuate seizure-associated behaviors in *repo > dube3a* flies, but can not completely suppress seizure behavior on their own (Roy et al., 2020).

Next, we examined whether vortioxetine or ketanserin altered the spontaneous seizure spike discharges in *repo > dube3a* flies. Consistent with the behavioral observations above, both vortioxetine or ketanserin feeding could reduce the occurrence of spontaneous burst spike discharges, and instead the spiking resembled grooming related activity observed in *repo > w*^*1118*^ (Fig. 5D). Specifically, vortioxetine-fed flies displayed a reduction in overall firing frequency and bursting at 0.4 μM, but not 0.04 μM (Fig. 5E & F). Ketanserin, displayed a stronger effect, with reductions in the overall firing frequency and bursting rate detectable at both concentrations. Indeed, the overall firing frequency for ketanserin-fed flies was not statistically distinct from *repo > w*^*1118*^ control flies, indicating a near-complete reversal of the phenotype (*p* = 0.50 for 0.04 μM, *p* = 0.74 for 0.4 μM). These electrophysiological findings largely corroborate the behavioral findings above that SSRIs and 5-HT_2A_ antagonists can reduce seizure activity in flies overexpressing *dube3a* in glia.

## 4. Discussion

Epilepsy is a common comorbidity in individuals with Dup15q syndrome, and seizure control is considered a major unmet medical need in these patients (Conant et al., 2014). New animal models with face validity, i.e. that have spontaneous (unprovoked) seizures, will be essential to the development of new targeted anti-epileptics for individuals with Dup15q syndrome. Neuronal overexpression of *Ube3a* in mouse models of Dup15q syndrome recapitulate some aspects of the repetitive stereotypic behavior and defective social interaction characteristic of the disease (Copping et al., 2017; Takumi, 2011). However spontaneous seizure phenotypes have not been reported in these models. In flies, overexpression of *dube3a* in glia, but not neurons, causes bang-sensitive hyperexcitable phenotypes (Hope et al., 2017; Roy et al., 2020). Indeed, in the context of other epilepsy syndromes, there is growing appreciation of the role of glia in contributing to associated pathophysiology (Eyo et al., 2017; Patel et al., 2019; Steinhauser et al., 2012; Tian et al., 2005). Here, we provide new behavioral and electrophysiological evidence of the critical role of glia in driving *dube3a*-associated seizure phenotypes in Drosophila. Importantly, this report provides the first documentation of spontaneous and recurrent seizure activity in any *UBE3A* overexpression model.

Although we found both neuronal- and glial-driven overexpression of *dube3a* led to detectable survival and motor phenotypes (Fig. 1), *repo > dube3a* flies displayed marked seizure-associated behavioral phenotypes evoked by high temperature stress (Figs. 2 & 3). Furthermore, electrophysiological analysis revealed most *repo > dube3a* flies showed recurrent and synchronized spike burst discharges indicative of spontaneous seizure activity (Fig. 4). This abnormal spiking bursting was not observed in flies overexpressing *dube3a* in neurons. Lastly, we studied the effect of two previously identified compounds which attenuate bang-sensitivity in *repo > dube3a* flies (Roy et al., 2020). As shown in Fig. 5, in *repo > dube3a* flies, we found ketanserin (a 5-HT_2A_ antagonist) and vortioxetine (a SSRI) reduce both the vulnerability to high temperature stress and the occurrence of spontaneous spike discharges. Together, these findings highlight the important role of glia in driving Dup15q-related seizure phenotypes and provide a road map for developing therapeutic strategies for the syndrome.

### 4.1. An automated approach to quantifying gliopathic seizure-related behavior in Drosophila

Like other Drosophila models of epilepsy, *repo > dube3a* transgenic flies are particularly amenable for high-throughput behavioral screens to identify genetic or pharmacological conditions that suppress seizure phenotypes. Two widely used methods to study seizure-associated behaviors in Drosophila are bang-sensitivity and high-temperature sensitivity assays, where observers score hyperexcitable behaviors following mechanical or temperature stress respectively (Benzer, 1973; Burg and Wu, 2012; Ganetzky and Wu, 1982; Melom and Littleton, 2013; Sun et al., 2012). Our approach, which combines automated fly tracking with machine learning to identify seizure-related events, makes several improvements to these established methods. First, automated fly tracking eliminates human scoring as a source of variability in the analysis. Second, the system requires fewer personnel costs, as the control of camera and stage temperature are automated. Finally, videos of fly behavior are available for quantifiable *post-hoc* analysis of behavioral features well beyond the initial study.

Automated video tracking approaches have been employed to characterize walking behavior in several hyperexcitable mutants (Chi et al., 2019; Iyengar et al., 2012; Kaas et al., 2016; Stone et al., 2013). Here, we built upon this work to identify seizure-related behavioral events based on locomotion tracking using a custom machine-learning approach. Several studies have employed hidden Markov models (HMM) like the one used here to classify behavioral states in animals (e.g. Wiggin et al., 2020; Jiang et al., 2019). Hidden Markov model strategies are also widely employed to detect seizure events based on EEG time-series data (Abdullah et al., 2012; Baldassano et al., 2016; Lee et al., 2018; Wong et al., 2007). We found the hidden Markov Classifier (HMC) classification was largely consistent with manual observations of immobilization behavior (Fig. 3C & D vs. Fig. 3E). Importantly the HMC provided classification on a frame-by-frame basis facilitating quantification of seizure behavior at a higher temporal resolution than the previous approach. Thus, drug-induced phenotypic variations can be quantitatively compared with each other (Fig. 5A-B).

Despite the utility of the HMC in this study, we recognize several potential limitations in our implementation. First, the specific parameters generated for the HMC are likely specific to identifying immobilization events in *repo > dube3a* flies, as the training dataset consisted of tracks from *repo > dube3a, repo > w^1118^, nsyb > dube3a,* and *nsyb > w^1118^* flies. Future studies could optimize the model by using a larger and more diverse data set of flies exhibiting high temperature-evoked seizure-related behaviors. Indeed, there are many Drosophila mutants which display heat induced seizures, and there are likely many subtle differences among the behavioral phenotypes displayed by these mutants. Second, our model designates ‘immobilization’ as the only seizure-related behavioral state. More sophisticated classifiers building on our approach may be used to designate the ‘spinning’ and ‘wing-buzz’ that also correspond to seizure-related behavior repertoire (Supplemental Video 2). Several behavioral classification approaches utilizing high-resolution images of fly posture (e.g. Berman et al., 2016; Mueller et al., 2019; Pereira et al., 2022) may better capture these events for high resolution classification.

### 4.2. Electrophysiological signatures of seizure activity in dube3a overexpressing flies

Recording from DLM flight muscles of tethered flies is a convenient electrophysiological readout of seizure activity in Drosophila (Iyengar and Wu, 2014; Lee and Wu, 2002; Pavlidis and Tanouye, 1995). During seizures, Drosophila flight muscles display characteristic bursts that are synchronized between the left and right motor units (Lee et al., 2019). These bursts present as self-similar loops in plots of the instantaneous firing frequency versus the instantaneous coefficient of variation (Fig. 4D, Lee et al., 2019). Previous studies have uncovered large-amplitude brain local field potential (LFP) signals which coincide with bursting activity. Our recordings revealed glial overexpression of *dube3a,* but not neuronal overexpression of *dube3a* leads to spontaneous seizures, observed as synchronized spike bursts (Fig. 4B). Approximately 50 % of *repo > dube3a* flies display spontaneous seizure bursts, while only a few *nsyb > dube3a* flies and no *repo > w^1118^* or *nsyb > w^1118^* flies showed this seizure activity (Fig. 4D, E, G). Flies that did not seize, instead displayed normal grooming-related spiking patterns. To directly demonstrate that the observed spike discharges in *repo > dube3a* flies originate from the central nervous system (rather than the neuromuscular junction), we injected mecamylamine, a blocker of central excitatory neurotransmission. Mecamylamine reliably blocked spike discharges indicating the activity is generated centrally (Fig. 4H). Together, these observations suggest *repo > dube3a* flies model critical aspects of hyperexcitability observed in Dup15q patients, that of spontaneous seizure activity.

Although *repo > dube3a* represents the first case of glial disfunction leading to spontaneous and recurrent seizures in flies, glial pathophysiology has been implicated in hyperexcitable phenotypes characteristic of several mutant strains. Flies carrying glia-specific disruptions of the Na^+^/K^+^ ATPase gene *ATPα* (Palladino et al., 2003), focal adhesion kinase gene *FAK* (Ueda et al., 2008) or the NCKX gene *zydeco* (Melom and Littleton, 2013) all display bang-sensitivity and high-temperature immobilization phenotypes like *repo > dube3a* flies. However, unlike these mutants, many *repo > dube3a* flies also display spontaneous seizures at room temperature. Given the penetrance of immobilization at high temperature in *repo > dube3a* flies (Fig. 3F), we expect the spontaneous spike bursts to similarly be temperature-dependent. Future electrophysiological analyses could also further resolve similarities between glial *dube3a* overexpression and other glia-specific disruptions described above. Interestingly, the Na^+^/K^+^ ATPase pump encoded by *ATPα* was previously shown to be a potential substrate for Dube3a in flies (Jensen et al., 2013). Furthermore, *repo > dube3a* flies exhibit high concentrations of extracellular K^+^ compared to control flies (Hope et al., 2017). Thus, a sub-set of the *repo > dube3a* seizure phenotypes may be due to attenuated *ATPα* function. However, because *ATPα* loss-of-function mutants do not show spontaneous seizures at room temperature, it is likely that other ubiquitin substrates regulated by Dube3a in flies contribute to the development of seizures. Ongoing efforts to characterize proteomic changes associated with *dube3a* overexpression may uncover additional Dube3a substrates that facilitate phenotype expression in *repo > dube3a* flies.

### 4.3. A pipeline to identify anti-epileptic drugs for the treatment of Dup15q syndrome

A particular strength of Drosophila epilepsy models is the high-throughput nature of behavioral phenotyping. Screens for genetic factors or pharmacological compounds that modify fly seizure phenotypes are straightforward and cost-effective (e.g. Stilwell et al., 2006). Several prior efforts have established certain anti-epileptic drugs (AEDs) can reduce susceptibility to mechanical shock or accelerate recovery in bang-sensitive mutants (Kuebler and Tanouye, 2002). Fly epilepsy models also offer the opportunity to identify new compounds (Kasuya et al., 2019), natural products (Dare et al., 2020) or even repurposed drugs (Roy et al., 2020) which suppress seizure activity. Interestingly, AED compounds can be efficacious in certain contexts, but have no effect on other models. For example, studies on the Na^+^ channel mutant *Shudderer* revealed that milk lipids can suppress spontaneous seizures, but these same lipids have no significant effect on the spontaneous leg-shaking and neuromuscular excitability phenotypes in the K^+^ double-mutant *eag Sh* (Kasuya et al., 2019). This observation highlights the need to develop models of a variety of pathophysiological processes leading to epilepsy and related seizure disorders.

The drug modulation studies of seizures in *repo > dube3a* flies presented here was motivated by a prior unbiased screen of FDA-approved compounds that revealed several compounds that could suppress bang-sensitivity in this model (Roy et al., 2020). The Roy et al. screen indicated compounds affecting serotonergic or dopaminergic signaling could suppress seizure phenotypes, somewhat surprisingly since these compounds are typically used as anti-depressants, not anti-epileptics. Specifically, SSRIs, 5-HT_1A_ agonists or 5-HT_2A_ antagonists reduced the recovery time from mechanical shock in *repo > dube3a* flies significantly. In contrast, 5-HT_1A_ antagonists or 5-HT_2A_ agonists hindered recovery following seizure induction (Roy et al., 2020). In Fig. 5, we tested two serotonergic compounds, vortioxetine (an SSRI), and ketanserin (a 5-HT_2A_ antagonist), using the automated video tracking and tethered fly electrophysiological assays. Consistent with bang sensitivity assays in Roy et al., we found at high temperature, both drugs reduced time spent immobilized (Fig. 5B). In the tethered fly preparation, we found both drugs (0.4 μM) could reduce both overall flight muscle spiking as well as spike bursts (Fig. 5D-F). Notably, most drug-fed flies eventually displayed high-temperature induced immobilization, and in these flies spontaneous spike discharges were sometimes observed. Thus, it is conceivable future drug screening efforts will yield compounds which, in combination with SSRIs, could further reverse seizure-related phenotypes in the *repo > dube3a* overexpression model of Dup15q syndrome.

Here we have shown that our gliacentric Drosophila model of Dup15q epilepsy continues to be a robust tool for the evaluation of new compounds specifically targeted to this disorder, where pharmacoresistant epilepsy is a major factor in the quality of life of these individuals. Moreover, we used this model to develop a robust video tracking tool to detect and quantify seizure events that we then validated using head fixed electrophysiology. The implications of this work are broad given the large number of fly homologues to human epilepsy associated genes. We anticipate that applying the tools developed here to other fly epilepsy models will narrow the range of drugs for specific epilepsy treatments (i.e. personalized medicine) especially using the drug repurposing approach.

Supplementary data to this article can be found online at https://doi.org/10.1016/j.nbd.2024.106651.

## Supplementary Material

1

2

3

4

5

6

## Data Availability

Data will be made available on request.

## References

[R1] Abdullah MH, Abdullah JM, Abdullah MZ, 2012. Seizure detection by means of hidden Markov model and stationary wavelet transform of electroencephalograph signals. In: Proceedings of 2012 IEEE-EMBS international conference on biomedical and health informatics.

[R2] Angelman H., 1965. Puppet’children a report on three cases. Dev. Med. Child Neurol 7 (6), 681–688. 10.1111/j.1469-8749.2008.03035.x 18754889

[R3] Baldassano S, Wulsin D, Ung H, Blevins T, Brown M-G, Fox E, Litt B, 2016. A novel seizure detection algorithm informed by hidden Markov model event states. J. Neural Eng 13 (3), 036011. 27098152 10.1088/1741-2560/13/3/036011 PMC4888894

[R4] Battaglia A., 2008. The inv dup (15) or idic (15) syndrome (Tetrasomy 15q). Orphanet J. Rare Dis 3 (1), 30. 10.1186/1750-1172-3-30. 19019226 PMC2613132

[R5] Benzer S., 1973. Genetic dissection of behavior. Sci. Am 229 (6), 24–37. 4202065 10.1038/scientificamerican1273-24

[R6] Berman GJ, Bialek W, Shaevitz JW, 2016. Predictability and hierarchy in Drosophila behavior. Proc. Natl. Acad. Sci 113 (42), 11943–11948. 27702892 10.1073/pnas.1607601113 PMC5081631

[R7] Besson M, Martin JR, 2005. Centrophobism/thigmotaxis, a new role for the mushroom bodies in Drosophila. J. Neurobiol 62 (3), 386–396. 10.1002/neu.20111. 15547935

[R8] Burg MG, Wu C-F, 2012. Mechanical and temperature stressor–induced seizure-and-paralysis behaviors in drosophila bang-sensitive mutants. J. Neurogenet 26 (2), 189–197. 22716921 10.3109/01677063.2012.690011 PMC3398232

[R9] Chi W, Iyengar ASR, Albersen M, Bosma M, Verhoeven-Duif NM, Wu C-F, Zhuang X, 2019. Pyridox (am) ine 5′-phosphate oxidase deficiency induces seizures in Drosophila melanogaster. Hum. Mol. Genet 28 (18), 3126–3136. 10.1093/hmg/ddz143. 31261385 PMC6737294

[R10] Chi W, Iyengar ASR, Fu W, Liu W, Berg AE, Wu C-F, Zhuang X, 2022. Drosophila carrying epilepsy-associated variants in the vitamin B6 metabolism gene PNPO display allele- and diet-dependent phenotypes. Proc. Natl. Acad. Sci 119 (9) 10.1073/pnas.2115524119 e2115524119. PMC8892510 35217610

[R11] Conant KD, Finucane B, Cleary N, Martin A, Muss C, Delany M, Murphy EK, Rabe O, Luchsinger K, Spence SJ, Schanen C, Devinsky O, Cook EH, LaSalle J, Reiter LT, Thibert RL, 2014. A survey of seizures and current treatments in 15q duplication syndrome. Epilepsia 55 (3), 396–402. 10.1111/epi.12530. 24502430

[R12] Cook ELVLBL, Courchesne R, Lincoln A, Shulman C, Lord C, Courchesne E, 1997. Autism or atypical autism in maternally but not paternally derived proximal 15q duplication. Am. J. Hum. Genet 60 (4), 928–934. 9106540 PMC1712464

[R13] Copping NA, Christian SGB, Ritter DJ, Islam MS, Buscher N, Zolkowska D, Pride MC, Berg EL, LaSalle JM, Ellegood J, Lerch JP, Reiter LT, Silverman JL, Dindot SV, 2017. Neuronal overexpression of Ube3a isoform 2 causes behavioral impairments and neuroanatomical pathology relevant to 15q11.2-q13.3 duplication syndrome. Hum. Mol. Genet 26 (20), 3995–4010. 10.1093/hmg/ddx289. 29016856 PMC5886211

[R14] Dare SS, Merlo E, Rodriguez Curt J, Ekanem PE, Hu N, Berni J, 2020. Drosophila Para (bss) flies as a screening model for traditional medicine: anticonvulsant effects of Annona senegalensis. Front. Neurol 11, 606919 10.3389/fneur.2020.606919. 33519685 PMC7838503

[R15] Depienne C, Moreno-De-Luca D, Heron D, Bouteiller D, Gennetier A, Delorme R, Chaste P, Siffroi J-P, Chantot-Bastaraud S, Benyahia B, Trouillard O, Nygren G, Kopp S, Johansson M, Rastam M, Burglen L, Leguern E, Verloes A, Leboyer M, Betancur C, 2009. Screening for genomic rearrangements and methylation abnormalities of the 15q11-q13 region in autism Spectrum disorders. Biol. Psychiatry 66 (4), 349–359. 10.1016/j.biopsych.2009.01.025. 19278672

[R16] DiStefano C, Gulsrud A, Huberty S, Kasari C, Cook E, Reiter LT, Thibert R, Jeste SS, 2016. Identification of a distinct developmental and behavioral profile in children with Dup15q syndrome. J. Neurodev. Disord 8, 1–13. 27158270 10.1186/s11689-016-9152-y PMC4858912

[R17] Eyo UB, Murugan M, Wu LJ, 2017. Microglia-neuron communication in epilepsy.Glia 65 (1), 5–18. 10.1002/glia.23006. 27189853 PMC5116010

[R18] Farook MF, DeCuypere M, Hyland K, Takumi T, LeDoux MS, Reiter LT, 2012. Altered serotonin, dopamine and norepinepherine levels in 15q duplication and Angelman syndrome mouse models. PLoS One 7 (8), e43030. 10.1371/journal.pone.0043030. 22916201 PMC3420863

[R19] Ferdousy F, Bodeen W, Summers K, Doherty O, Wright O, Elsisi N, Hilliard G, O’Donnell JM, Reiter LT, 2011. Drosophila Ube3a regulates monoamine synthesis by increasing GTP cyclohydrolase I activity via a non-ubiquitin ligase mechanism. Neurobiol. Dis 41 (3), 669–677 doi:S0969-9961(10)00394-3[pii]0.1016/j.nbd.2010.12.001. 21147225 10.1016/j.nbd.2010.12.001 PMC3040417

[R20] Ganetzky B, Wu C-F, 1982. Indirect suppression involving behavioral mutants with altered nerve excitability in Drosophila melanogaster. Genetics 100 (4), 597–614. 17246073 10.1093/genetics/100.4.597 PMC1201835

[R21] Hope KA, LeDoux MS, Reiter LT, 2017. Glial overexpression of Dube3a causes seizures and synaptic impairments in Drosophila concomitant with down regulation of the Na+/K+ pump ATPα. Neurobiol. Dis 108, 238–248. 10.1016/j.nbd.2017.09.003. 28888970 PMC5675773

[R22] Iyengar A, Wu C-F, 2014. Flight and seizure motor patterns in Drosophila mutants: simultaneous acoustic and electrophysiological recordings of wing beats and flight muscle activity. J. Neurogenet 28 (3–4), 316–328. 10.3109/01677063.2014.957827. 25159538 PMC5555410

[R23] Iyengar A, Wu C-F, 2021. Fly seizure EEG: field potential activity in the Drosophila brain. J. Neurogenet 35 (3), 295–305. 34278939 10.1080/01677063.2021.1950714 PMC10012387

[R24] Iyengar A, Imoehl J, Ueda A, Nirschl J, Wu C-F, 2012. Automated quantification of locomotion, social interaction, and mate preference in Drosophila mutants. J. Neurogenet 26 (3–4), 306–316. 23106154 10.3109/01677063.2012.729626 PMC3613147

[R25] Iyengar A, Ruan H, Wu C-F, 2022. Distinct aging-vulnerable and-resilient trajectories of specific motor circuit functions in oxidation-and temperature-stressed Drosophila. Eneuro 9 (1). 10.1523/ENEURO.0443-21.2021 PMC8805199 34876473

[R26] Jensen L, Farook MF, Reiter LT, 2013. Proteomic profiling in Drosophila reveals potential Dube3a regulation of the actin cytoskeleton and neuronal homeostasis. PLoS One 8 (4), e61952. 10.1371/journal.pone.0061952. 23626758 PMC3633955

[R27] Jiang Z, Crookes D, Green BD, Zhao Y, Ma H, Li L, Zhang S, Tao D, Zhou H, 2019. Context-aware mouse behavior recognition using hidden Markov models. IEEE Trans. Image Process 28 (3), 1133–1148. 10.1109/tip.2018.2875335. 30307863

[R28] Kaas GA, Kasuya J, Lansdon P, Ueda A, Iyengar A, Wu C-F, Kitamoto T, 2016. Lithium-responsive seizure-like hyperexcitability is caused by a mutation in the Drosophila voltage-gated sodium channel gene paralytic. Eneuro 3 (5). 10.1523/ENEURO.0221-16.2016 PMC5103163 27844061

[R29] Kasuya J, Iyengar A, Chen H-L, Lansdon P, Wu C-F, Kitamoto T, 2019. Milkwhey diet substantially suppresses seizure-like phenotypes of paraShu, a Drosophila voltage-gated sodium channel mutant. J. Neurogenet 33 (3), 164–178. 31096839 10.1080/01677063.2019.1597082 PMC6641994

[R30] Kishino T, Lalande M, Wagstaff J, 1997. UBE3A/E6-AP mutations cause Angelman syndrome. Nat. Genet 15 (1), 70–73. 10.1038/ng0197-70. 8988171

[R31] Krishnan V, Stoppel DC, Nong Y, Johnson MA, Nadler MJS, Ozkaynak E, Teng BL, Nagakura I, Mohammad F, Silva MA, Peterson S, Cruz TJ, Kasper EM, Arnaout R, Anderson MP, 2017. Autism gene Ube3a and seizures impair sociability by repressing VTA Cbln1. Nature 543 (7646), 507–512. 10.1038/nature21678. 28297715 PMC5364052

[R32] Kuebler D, Tanouye M, 2002. Anticonvulsant valproate reduces seizure-susceptibility in mutant Drosophila. Brain Res. 958 (1), 36–42. 10.1016/s0006-8993(02)03431-5. 12468028

[R33] Lalande M., 1996. PARENTAL IMPRINTING AND HUMAN DISEASE. Annu. Rev. Genet 30 (1), 173–195. 10.1146/annurev.genet.30.1.173. 8982453

[R34] LaSalle J, Reiter LT, Chamberlain SJ, 2015. Epigenetic regulation of UBE3A and roles in human neurodevelopmental disorders. Epigenomics 7 (7), 1213–1228. 26585570 10.2217/epi.15.70 PMC4709177

[R35] Lee J, Wu C-F, 2002. Electroconvulsive seizure behavior in Drosophila: analysis of the physiological repertoire underlying a stereotyped action pattern in bang-sensitive mutants. J. Neurosci 22 (24), 11065–11079. 12486202 10.1523/JNEUROSCI.22-24-11065.2002 PMC6758420

[R36] Lee M, Youn I, Ryu J, Kim D-H, 2018. Classification of both seizure and non-seizure based on EEG signals using hidden Markov model. In: 2018 IEEE international conference on big data and smart computing (BigComp).

[R37] Lee J, Iyengar A, Wu C-F, 2019. Distinctions among electroconvulsion-and proconvulsant-induced seizure discharges and native motor patterns during flight and grooming: quantitative spike pattern analysis in Drosophila flight muscles. J. Neurogenet 33 (2), 125–142. 30982417 10.1080/01677063.2019.1581188 PMC6602807

[R38] Lopez SJ, Segal DJ, LaSalle JM, 2019. UBE3A: an E3 ubiquitin ligase with genome-wide impact in neurodevelopmental disease [Mini review]. Front. Mol. Neurosci 11 10.3389/fnmol.2018.00476. PMC6338038 30686997

[R39] Matsuura T, Sutcliffe JS, Fang P, Galjaard R-J, Jiang Y-H, Benton CS, Rommens JM, Beaudet AL, 1997. De novo truncating mutations in E6-AP ubiquitin-protein ligase gene (UBE3A) in Angelman syndrome. Nat. Genet 15 (1), 74–77. 8988172 10.1038/ng0197-74

[R40] Melom JE, Littleton JT, 2013. Mutation of a NCKX eliminates glial microdomain calcium oscillations and enhances seizure susceptibility. J. Neurosci 33 (3), 1169–1178. 23325253 10.1523/JNEUROSCI.3920-12.2013 PMC3600868

[R41] Mueller JM, Ravbar P, Simpson JH, Carlson JM, 2019. Drosophila melanogaster grooming possesses syntax with distinct rules at different temporal scales. PLoS Comput. Biol 15 (6), e1007105. 31242178 10.1371/journal.pcbi.1007105 PMC6594582

[R42] Nakatani J, Tamada K, Hatanaka F, Ise S, Ohta H, Inoue K, Tomonaga S, Watanabe Y, Chung YJ, Banerjee R, Iwamoto K, Kato T, Okazawa M, Yamauchi K, Tanda K, Takao K, Miyakawa T, Bradley A, Takumi T, 2009. Abnormal behavior in a chromosome-engineered mouse model for human 15q11-13 duplication seen in autism. Cell 137 (7), 1235–1246. 10.1016/j.cell.2009.04.024. 19563756 PMC3710970

[R43] Palladino MJ, Bower JE, Kreber R, Ganetzky B, 2003. Neural dysfunction and neurodegeneration in Drosophila Na+/K+ ATPase alpha subunit mutants. J. Neurosci 23 (4), 1276–1286. 10.1523/jneurosci.23-04-01276.2003. 12598616 PMC6742270

[R44] Patel DC, Tewari BP, Chaunsali L, Sontheimer H, 2019. Neuron-glia interactions in the pathophysiology of epilepsy. Nat. Rev. Neurosci 20 (5), 282–297. 10.1038/s41583-019-0126-4. 30792501 PMC8558781

[R45] Pavlidis P, Tanouye MA, 1995. Seizures and failures in the giant fiber pathway of Drosophila bang-sensitive paralytic mutants. J. Neurosci 15 (8), 5810–5819. 10.1523/jneurosci.15-08-05810.1995. 7643221 PMC6577637

[R46] Pereira TD, Tabris N, Matsliah A, Turner DM, Li J, Ravindranath S, Papadoyannis ES, Normand E, Deutsch DS, Wang ZY, 2022. SLEAP: A deep learning system for multi-animal pose tracking. Nat. Methods 19 (4), 486–495. 35379947 10.1038/s41592-022-01426-1 PMC9007740

[R47] Roy B, Han J, Hope KA, Peters TL, Palmer G, Reiter LT, 2020. An unbiased drug screen for seizure suppressors in duplication 15q syndrome reveals 5-HT(1A) and dopamine pathway activation as potential therapies. Biol. Psychiatry 88 (9), 698–709. 10.1016/j.biopsych.2020.03.021. 32507391 PMC7554174

[R48] Smith SEP, Zhou Y-D, Zhang G, Jin Z, Stoppel DC, Anderson MP, 2011. Increased Gene Dosage of *Ube3a* Results in Autism Traits and Decreased Glutamate Synaptic Transmission in Mice. Sci. Transl. Med 3 (103) 10.1126/scitranslmed.3002627, 103ra197–103ra197. PMC3356696 21974935

[R49] Steinhäuser C, Seifert G, Bedner P, 2012. Astrocyte dysfunction in temporal lobe epilepsy: K+ channels and gap junction coupling. Glia 60 (8), 1192–1202. 10.1002/glia.22313. 22328245

[R50] Stilwell GE, Saraswati S, Littleton JT, Chouinard SW, 2006. Development of a Drosophila seizure model for in vivo high-throughput drug screening. Eur. J. Neurosci 24 (8), 2211–2222. 10.1111/j.1460-9568.2006.05075.x. 17074045

[R51] Stone B, Evans L, Coleman J, Kuebler D, 2013. Genetic and pharmacological manipulations that alter metabolism suppress seizure-like activity in Drosophila. Brain Res. 1496, 94–103. 23247062 10.1016/j.brainres.2012.12.007

[R52] Sun L, Gilligan J, Staber C, Schutte RJ, Nguyen V, O’Dowd DK, Reenan R, 2012. A knock-in model of human epilepsy in Drosophila reveals a novel cellular mechanism associated with heat-induced seizure. J. Neurosci 32 (41), 14145–14155. 23055484 10.1523/JNEUROSCI.2932-12.2012 PMC3482260

[R53] Takumi T., 2011. The neurobiology of mouse models syntenic to human chromosome 15q. J. Neurodev. Disord 3 (3), 270–281. 10.1007/s11689-011-9088-1. 21789598 PMC3261275

[R54] Tian GF, Azmi H, Takano T, Xu Q, Peng W, Lin J, Oberheim N, Lou N, Wang X, Zielke HR, Kang J, Nedergaard M, 2005. An astrocytic basis of epilepsy. Nat. Med 11 (9), 973–981. 10.1038/nm1277. 16116433 PMC1850946

[R55] Ueda A, Grabbe C, Lee J, Lee J, Palmer RH, Wu CF, 2008. Mutation of Drosophila focal adhesion kinase induces bang-sensitive behavior and disrupts glial function, axonal conduction and synaptic transmission. Eur. J. Neurosci 27 (11), 2860–2870. 10.1111/j.1460-9568.2008.06252.x. 18540882 PMC2671471

[R56] Ueda A, Berg A, Khan T, Ruzicka M, Li S, Cramer E, Iyengar A, Wu C-F, 2023. Intense light unleashes male–male courtship behaviour in wild-type Drosophila. Open Biol. 13 (7), 220233 10.1098/rsob.220233. 37463658 PMC10353890

[R57] Urraca N, Cleary J, Brewer V, Pivnick EK, McVicar K, Thibert RL, Schanen NC, Esmer C, Lamport D, Reiter LT, 2013. The interstitial duplication 15q11. 2-q13 syndrome includes autism, mild facial anomalies and a characteristic EEG signature. Autism Res. 6 (4), 268–279. 23495136 10.1002/aur.1284 PMC3884762

[R58] Vu TH, Hoffman AR, 1997. Imprinting of the Angelman syndrome gene, UBE3A, is restricted to brain. Nat. Genet 17 (1), 12–13. 9288087 10.1038/ng0997-12

[R59] Wiggin TD, Goodwin PR, Donelson NC, Liu C, Trinh K, Sanyal S, Griffith LC, 2020. Covert sleep-related biological processes are revealed by probabilistic analysis in Drosophila. Proc. Natl. Acad. Sci 117 (18), 10024–10034. 32303656 10.1073/pnas.1917573117 PMC7211995

[R60] Williams CA, 2005. Neurological aspects of the Angelman syndrome. Brain and Development 27 (2), 88–94. 10.1016/j.braindev.2003.09.014. 15668046

[R61] Wong S, Gardner AB, Krieger AM, Litt B, 2007. A stochastic framework for evaluating seizure prediction algorithms using hidden Markov models. J. Neurophysiol 97 (3), 2525–2532. 17021032 10.1152/jn.00190.2006 PMC2230664

