# Supplementary material for: Glial expression of Drosophila UBE3A causes spontaneous seizures that can be modulated by 5-HT signaling
Source: Neurobiol Dis. Author manuscript; Available in PMC 2024 Dec 24. (PMC11668239; doi:10.1016/j.nbd.2024.106651)
Supplement: 4 [file NIHMS2022323-supplement-4.docx]

**Supplemental Figures**

**
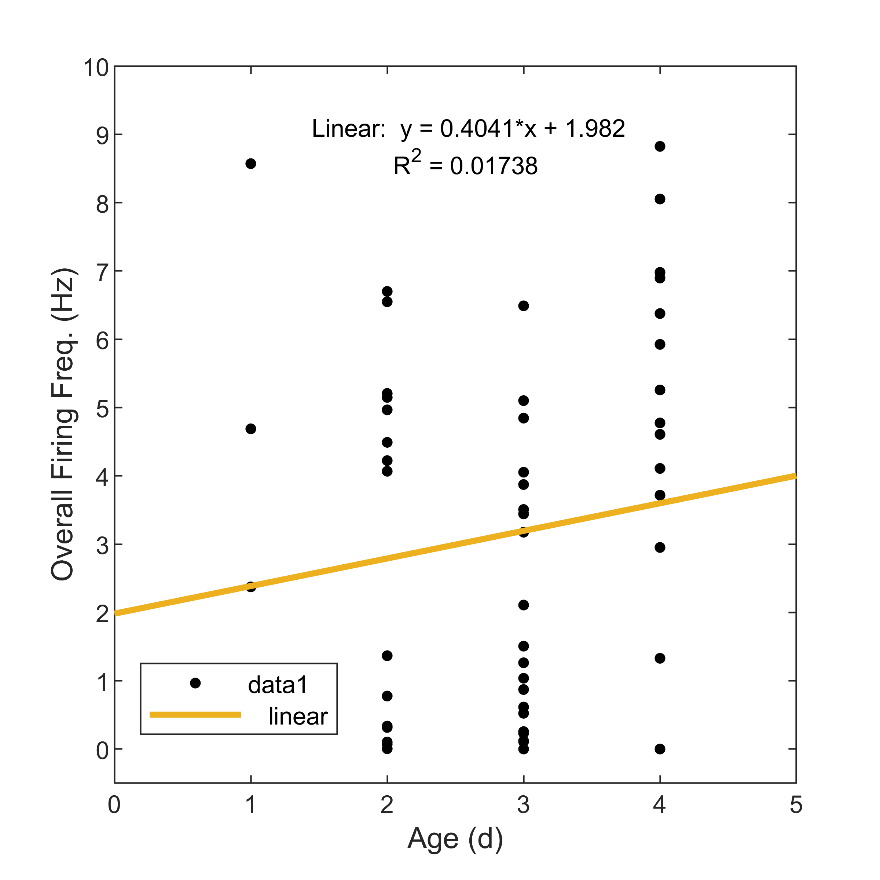
**

**Supplemental Figure 1. Overall DLM firing rate versus fly age.** Scatterplot of the data in Figure 4F plotted against fly age. Line indicates Pearson’s correlation coefficient (r^2^ value as indicated, p = 0.33).

**
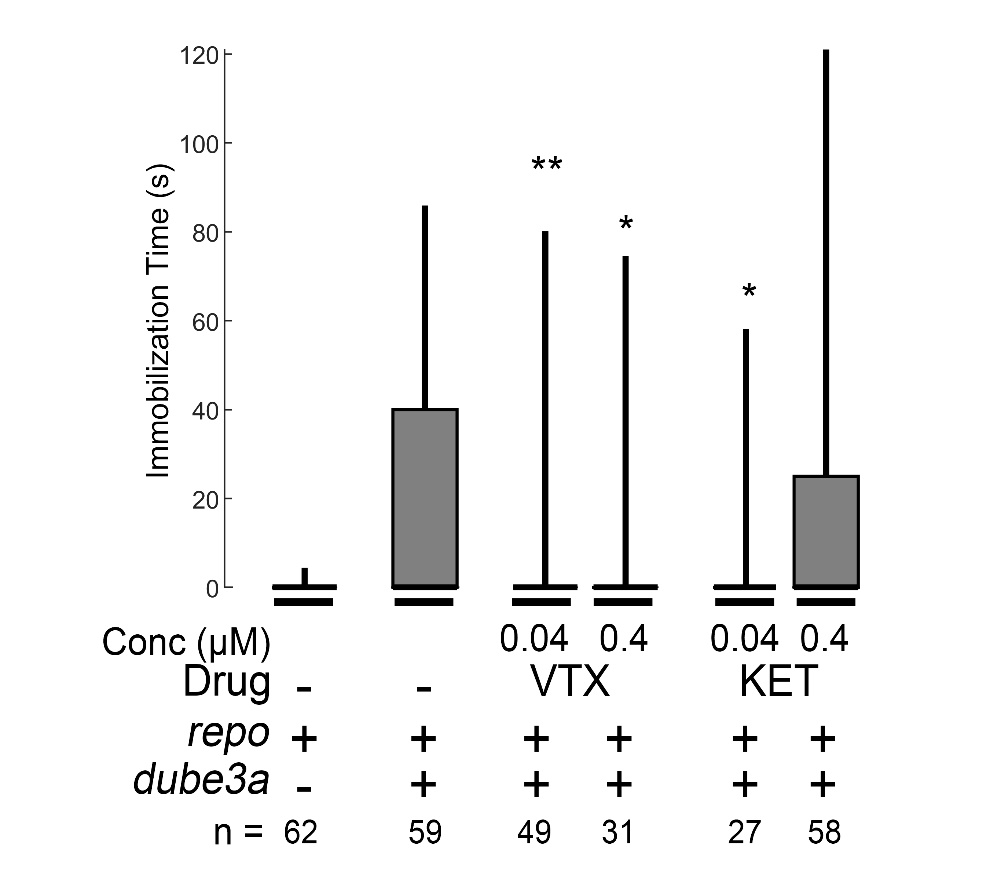
**

**Supplemental Figure 2. Immobilization in VTX and KET-fed *repo>dube3a* flies.** HMC-identified immobilization during the baseline video recording period.
