# Supplementary material for: Glial expression of Drosophila UBE3A causes spontaneous seizures that can be modulated by 5-HT signaling
Source: Neurobiol Dis. Author manuscript; Available in PMC 2024 Dec 24. (PMC11668239; doi:10.1016/j.nbd.2024.106651)
Supplement: 5 [file NIHMS2022323-supplement-5.docx]

***Supplemental Information - Behavioral Classifier Construction***

*Pre-Processing*

To prepare tracks for classifier analysis we first segmented tracks into 2-s coordinate trajectories consisting of 61 (**x**, **y**) coordinate pairs, i.e. **x** = x_0,_…,x_60_ and **y** = y_0,_…,y_60_. Successive 2-s trajectories overlapped with the previous trajectory by 1 s. Thus, for a 10-min video containing ~ 18,000 frames, there are ~600 trajectories per fly. To align trajectories, the initial values were subtracted from **x** and **y** such each trajectory started at the origin, i.e. (**x’**, **y’**) = (**x**-x_0_ , **y**-y_0_). These trajectories are then rotated such that they end in the same direction. The rotation angle is θ = atan (x_60_’ / y_60_’), and the aligned coordinates (**x”**,**y”**) = (**x’**cos (θ) + **y’**sin (θ) , **‑x’**sin (θ) + **y’**cos (θ))

The aligned coordinate trajectories, (**x”**,**y”**) are used for all subsequent analyses (S Figure XA-C). Thus for each of the ~600 trajectories corresponding to a single fly’s activity during the 10-min video, we have a single 61 x 2 matrix (**x”** , **y”**).

To decompose trajectories into principal components, we created a matrix, **CT**, representing all trajectories from *repo* > *w^1118^* (n = 138 flies, 74,520 trajectories) and *repo > dube3a* flies studied (n = 115 flies, 62,100 trajectories). Both males and females were used in this analysis. We created this data set by transforming each of the *i =* 136,620 coordinate trajectory into a 1 x 122 array, **ct**_i_ **=** (**x”^T^** , **y”^T^**). Each row of **CT** is coordinate trajectory **ct**_i_. Thus, the coordinate trajectories from a single fly are represented by ~ 600 rows in **CT.** In total **CT** has 136,620 total rows, 62,100 rows from *repo > dube3a* flies, and 74,520 from *repo > w^1118^* flies. We then performed principal component analysis on matrix **CT**, using the MATLAB function ‘pca’. The first four principal components (eigenvectors) corresponded with modes of locomotion (forward movement, turning, forward jitter and turning jitter), with weights (eigenvalues) corresponding with relative variation in the data set associated. Because most variation was captured by the first principal component (78%), we used this component in constructing HMM classifier. Thus the 136,620 x 122 matrix **CT** can be reduced to a forward movement vector, **fm,** a 136,620 x 1 array, with each value representing forward movement for a single trajectory.

*HMM Classifier Construction*

We created a HMM classifier that determined whether a fly was in one of three “hidden” states, walking, pausing, immobilized based on the “directly” observed parameter of forward movement (**fm**). We found a histogram of **fm** had two modes corresponding to forward movement or no forward movement (with a cutoff at -11 units). To find the state transition and emission matrices of the HMM, we used the Baum-Welch algorithm, implemented in MATLAB using the ‘hmmtrain’ function. The initial transition matrix was estimated as follows:

$$\begin{matrix} & to walking & to pausing & to immobilization \\ \mathrm{walking} & 0.855 & 0.135 & 0.010 \\ \mathrm{pausing} & 0.266 & 0.724 & 0.010 \\ \mathrm{immobilization} & 0.010 & 0.010 & 0.980 \end{matrix}$$

These values were based on the proportion of time *repo>w^1118^* flies displayed forward movement (65%) versus no forward movement (35%), with a low (1%) probability of transitioning to immobilization. The initial emission matrix was:

$$\begin{matrix} & \mathrm{low}\mathbf{fm} & \mathrm{high}\mathbf{fm} \\ \mathrm{walking} & 0.18 & 0.82 \\ \mathrm{pausing} & 0.75 & 0.25 \\ \mathrm{immobilization} & 0.95 & 0.05 \end{matrix}$$

With these initial parameters, the Baum-Welch algorithm reliably converged on optimum solution. The optimal transition matrix was:

$$\begin{matrix} & to walking & to pausing & to immobilization \\ \mathrm{walking} & 0.918 & 0.082 & 0.000 \\ \mathrm{pausing} & 0.198 & 0.774 & 0.028 \\ \mathrm{immobilization} & 0.002 & 0.018 & 0.980 \end{matrix}$$

While the optimal emission matrix was:

$$\begin{matrix} & \mathrm{low}\mathbf{fm} & \mathrm{high}\mathbf{fm} \\ \mathrm{walking} & 0.067 & 0.933 \\ \mathrm{pausing} & 0.865 & 0.135 \\ \mathrm{immobilization} & 0.994 & 0.006 \end{matrix}$$

We found that varying the initial values of the emission or transition matrix by up to 20% did not alter the convergence to these optimal values.

*HMM decoding*

To classify trajectories as likely walking, pausing or immobilization based on trajectories, we applied the MATLAB ‘hmmdecode’ function. The inputs to this function are the observed **fm** array, and the Transition and Emission matrices obtained following the Baum-Welch algorithm above.
